# Induction of gametocytogenesis in human malaria parasites: from stress to genome editing

**DOI:** 10.3389/fmicb.2025.1688506

**Published:** 2025-10-22

**Authors:** Rishitharan Subramaniam, Zi Yan Chiew, Nabel Darwish binti Zuhaidi, Yee Ling Lau, Fei Wen Cheong

**Affiliations:** Department of Parasitology, Faculty of Medicine, Universiti Malaya, Kuala Lumpur, Malaysia

**Keywords:** *Plasmodium*, gametocytes, malaria, genome engineering, gametocytogenesis induction, genome editing

## Abstract

Gametocytogenesis is a crucial process in which malaria parasites transition from their asexual stage to the sexual-stage gametocytes. This transformation enables the parasite to infect and multiply within the *Anopheles* mosquito, the vector responsible for transmitting the disease between hosts. Understanding how gametocytogenesis works and how it can be controlled offer insights into the malaria life cycle and potential strategies for controlling the transmission. Significant efforts have been dedicated to gametocytogenesis induction in the laboratory using *in vitro* parasite cultures over the past few decades. This mini review aims to summarize the various gametocytogenesis induction methods employed thus far in human *Plasmodium* species, moving from conventional means of environmental stressors to the cutting-edge technology of genome editing to achieve precise modifications on various sexual conversion-related genes. While massive strides have been made in both domains of gametocytogenesis induction methods, the scalability of gametocytogenesis induction leaves much to be desired, especially for non *falciparum* species. In conclusion, integrating knowledge from both approaches is crucial to developing highly efficient methods for inducing gametocytogenesis in *Plasmodium* spp. This integration can enhance our understanding of the processes involved in gametocytogenesis and support the search for novel strategies with potential implications for malaria control and eradication.

## 1 Introduction

Despite malaria’s first documentation back in 500 B.C.E, humanity has yet to develop a high efficacy vaccine for this disease (Palatnik-de-Sousa and Nico, 2020). In 2023, there were approximately 263 million cases worldwide (World Health Organization, 2024); Malaria parasites are able to infect the female *Anopheles* mosquito vector after it takes a blood meal from a host infected with malaria, spreading the disease when the infected vector takes a different blood meal from a different host. In humans the five significant species are *Plasmodium falciparum, Plasmodium ovale, Plasmodium malariae, Plasmodium vivax* and *Plasmodium knowlesi* (Sato, 2021). Gametocytes are the sole stage of *Plasmodium* species capable of transmission to mosquitoes, wherein it matures into sporozoites within the infected female mosquitoes thus contributing to infection of the host (Ngotho et al., 2019). As malaria parasites become increasingly resistant to widely used schizonticidal drugs, transmission-blocking strategies have attained prominence in malaria control efforts, with gametocytes emerging as promising targets for new drug development (Kumar et al., 2018; Wadi et al., 2020). Conventional antimalarials are effective against asexual stages and immature gametocytes, but they have limited impact on mature gametocytes, which are crucial for transmission (Baker, 2010; Portugaliza et al., 2020). Looking into vaccine development, *P. falciparum* sporozoite (PfSPZ) formulations by Sanaria Inc. have demonstrated moderate protection in clinical trials using radiated-, chemo-, or genetically attenuated sporozoites (Bijker et al., 2015; Duffy, 2022). In order to obtain these sporozoites, mosquito infections using gametocytes need to be carried out. A more recent study has also shown to expose a vulnerability in the sporozoite stage of the parasite by targeting a previously cryptic epitope on the malaria parasite’s circumsporozoite protein (PfCSP), allowing the most potent antibody in this group, MAD21-101, provide complete protection against *P. falciparum* infection in a humanized mouse model (Dacon et al., 2025). All these studies stress the importance of large-scale gametocyte production to acquire sufficient study materials.

## 2 Gametocytogenesis and its regulation

The decision to become a gametocyte appears to be made during the asexual cycle, prior to the formation of gametocytes. The sexual differentiation necessary for transmission starts when asexual parasites commit to gametocytogenesis (Baker, 2010). This sexually committed stage, which deterministically leads to sexual development subsequently, is identified by the expression of PfAP2-G. Sexual conversion then follows commitment, and present evidence suggests that this can occur through either same-cycle conversion or next-cycle conversion, both of which result in sexual ring stages that eventually develop into gametocytes (Portugaliza et al., 2019). In *P. falciparum*, low quantities of gametocytes usually appear in peripheral blood around the 10–12th day following the onset of fever and continue to rise during the next 2–3 weeks (Shute and Maryon, 1951; Gautam et al., 2020). On the contrary, *P. knowlesi* gametocytes mature quickly within 1.5–2 days, and have a remarkably short lifespan in the bloodstream with viability ranging 5–12 h before degenerating (Hawking et al., 1968; Carter et al., 1988; Amir, 2016). *P. vivax* gametocytes arise early in infection, appearing within 3 days after the emergence of asexual parasites whereas *P. malariae* gametocyte production is most likely to begin during the early stages of intraerythrocytic asexual replication (Bousema and Drakeley, 2011; Fatmaningsih et al., 2024). In *P. ovale*, gametocyte formation begins continuously with the early rounds of intraerythrocytic asexual reproduction and appears in the peripheral blood slightly earlier than in benign tertian malaria (Shute and Maryon, 1951). The gametocytes take about 5 days to mature, although their lifespan remains unknown (Amir, 2016).

Zooming down to the molecular mechanism behind gametocytogenesis reveals that at the core of the sexual commitment switch in *P. falciparum* is the transcription factor AP2-G, a member of the ApiAP2 family, which has been associated with the positive regulation of sexual commitment and regulates over 400 genes involved in the early stages of gametocytogenesis. The expression of AP2-G is tightly linked to gametocyte formation and maturation, and disruption of this gene results in a complete block of gametocytogenesis (Kafsack et al., 2014; Sinha et al., 2014). Among the genes notably upregulated during PfAP2-G overexpression are *msrp1*, *GEXP05*, and *Pfs16*. An experimental parasites line lacking *msrp1* (Δ*msrp1*) exhibit a slightly elevated commitment rate, suggesting that while it serves as a marker of sexually committed parasites, however, it is not essential for inducing or driving gametocyte formation (Josling et al., 2020). Although GEXP05 and Pfs16 are expressed as early as the sexual ring stage, its transcripts are insufficiently specific as markers for sexual parasites (Portugaliza et al., 2019; Singh et al., 2021). In contrast, another gene *GEXP02* was identified as a valuable early gametocyte marker and a reliable indicator of sexual conversion (Portugaliza et al., 2019). Under normal asexual conditions, the *ap2-g* locus is embedded in heterochromatin, marked by H3K9me3 and bound by heterochromatin protein 1 (HP1), maintaining it in a silenced state, and this heterochromatin-based silencing prevents the premature or unnecessary activation of gametocyte development, ensuring that most parasites remain in a proliferative asexual cycle (Brancucci et al., 2014; Bui Hai et al., 2021). In *P. falciparum*, the GDV1 (Gametocyte Development 1) protein acts upstream in this regulatory hierarchy (Filarsky et al., 2018). GDV1 binds to heterochromatic regions and physically interacts with HP1, triggering HP1 eviction specifically from the *ap2-g* locus. This chromatin remodeling, which is the ATP-driven reorganization of nucleosomes that opens compacted DNA (Phillips and Shaw, 2008) derepresses *ap2-g*, allowing its transcription and subsequent expression of PfAP2-G protein. Environmental cues such as nutrient availability influence GDV1 expression, and an antisense RNA regulates its levels, providing a layer of responsiveness to external triggers (Rea et al., 2018). Once expressed, PfAP2-G initiates a positive feedback loop by binding its own promoter and upregulating early gametocyte genes, solidifying sexual commitment. However, while *ap2-g* is essential for commitment and early gametocytogenesis, its necessity for later stages, such as maturation to stage V, has yet to be clarified (Josling et al., 2020).

**FIGURE 1:**
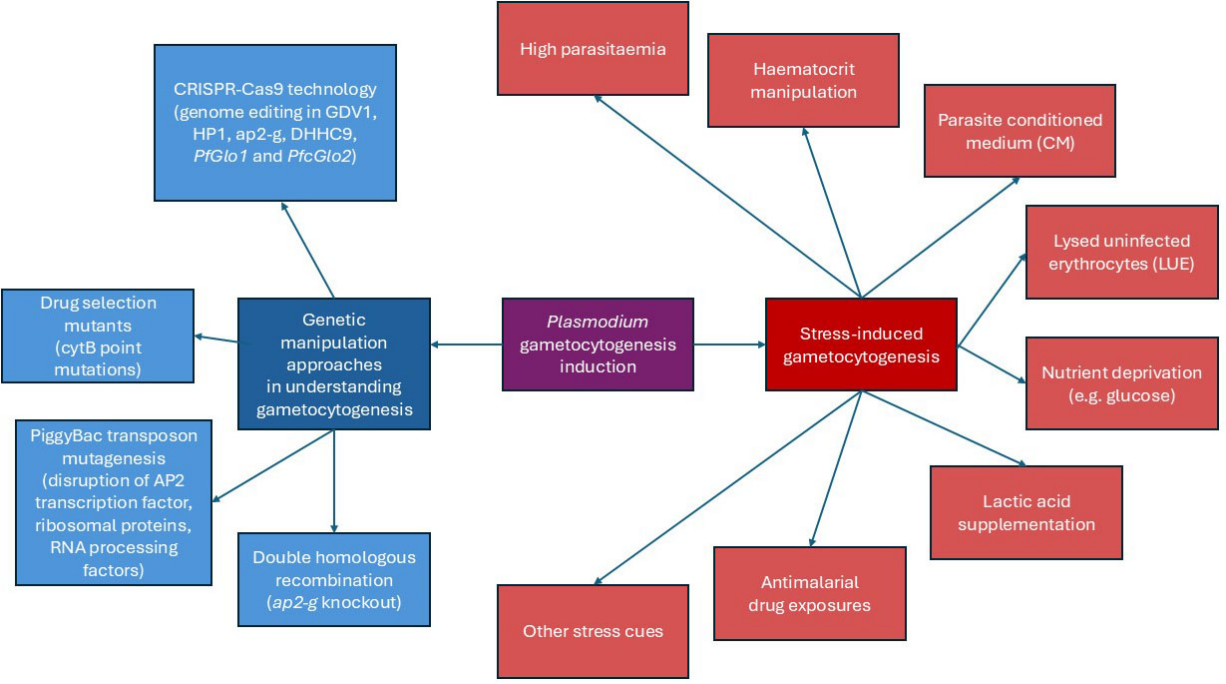
Schematic diagram of *Plasmodium* gametocytogenesis induction methods.

As gametocytogenesis plays a critical role in the transmission of parasites into the mosquito vector, understanding and controlling gametocytogenesis is essential not only for interrupting the transmission cycle but also for developing targeted strategies aimed at reducing the spread of malaria. The next sections summarize the various gametocytogenesis induction methods focusing on stress-based methods and genetic manipulation approaches employed in human *Plasmodium* species to understand the gametocytogenesis mechanisms thus far (Figure 1).

## 3 Stress-induced gametocytogenesis

*Plasmodium falciparum* accounts for over 90% of global malaria cases and is responsible for the majority of malaria-related deaths (World Health Organization, 2024). It is also one of the two only human-infecting *Plasmodium* species that can be continuously cultured *in vitro. P. falciparum* was first cultured continuously *in vitro* by Trager and Jensen (1976), using human red blood cells and serum in RPMI 1640 medium under specific gas conditions (low oxygen and high carbon dioxide) (Trager and Jensen, 1976). Then, *P. knowlesi in vitro* culture was established in 2010s adopting the similar protocol, thus making it the primary focus of most laboratory-based research.

A basic prerequisite for studying gametocyte biology and developing drugs and vaccines is the availability of healthy, viable, and reproducible gametocytes *in vitro* (Omorou et al., 2022). Most *in vitro* research to date has used environmental stressors to trigger gametocytogenesis, mainly targeting *P. falciparum*. These stressors include nutrient deprivation, sudden drops in haematocrit, conditioned media from parasite cultures that contain metabolites and secreted stress signals, elevated parasitaemia, and adjuvants that mimic the physiological conditions found in the host. Laboratory research involving *P. vivax* commonly requires the use of infected non-human primates owing to the inability to continuously culture the parasite *in vitro* (Galinski and Barnwell, 2008; Gunalan et al., 2020). Meanwhile, *P. knowlesi*’s asexual phases have been successfully modified for *in vitro* culture in human and macaque erythrocytes (Moon et al., 2013; Armistead et al., 2018). Nevertheless, researchers have yet to report efficient methods for gametocyte production in *P. knowlesi*. To date, only Armistead et al. (2018) have achieved gametocyte production using a *P. knowlesi in vitro* culture strain along with sporadic, low-level mosquito infectivity in transmission study.

By mimicking the physiological conditions of the host, which are characterized by elevated stress levels, high parasitaemia *in vitro* (8% or higher) promotes sexual development over asexual forms (Price et al., 1999; Buchholz et al., 2011). When combined with other stressors, such as lysed uninfected erythrocytes (LUE), parasite-conditioned medium, nutrient starvation, and limiting fresh erythrocytes, the impact of elevated parasitaemia on gametocytogenesis is further amplified (Ifediba and Vanderberg, 1981; Omorou et al., 2022). Alternatively, transient reduction of haematocrit during culture has been reported to indirectly facilitate increased parasitaemia. Bounkeua et al. (2010) used high parasitaemia ranging from 8%–15% to initiate gametocyte cultures, with 8%–10% producing the greatest yields of gametocytes. Conversely, Carter and Miller (1979) found that diluting cultures to lower parasitaemia at 0.1% reduced the rate of conversion to gametocytes, which then rose after several days of growth in culture.

As indicated in Table 1, both Buchholz et al. (2011) and Reader et al. (2015) discovered that lowering haematocrit enhances gametocytogenesis, however the outcomes varied with the accompanying stressor. Buchholz et al. (2011) found slight increases in sexual stages (∼ 0.15%) when combined with antimalarial exposure, while Reader et al. (2015) obtained much higher gametocytaemia under nutrient deprivation (∼ 5.1%). This implies that haematocrit drop alone is insufficient, but acts synergistically with other stress cues to promote sexual commitment. Alternatively, high parasitaemia can also be achieved via the crash method, a traditional technique where asexual parasitaemia progressively rises without the addition of fresh erythrocytes. This leads to nutrient stress, which causes the asexual population collapse thereby triggering gametocytogenesis as a survival mechanism (Ifediba and Vanderberg, 1981; Fivelman et al., 2007; Saliba and Jacobs-Lorena, 2013; Tripathi et al., 2020). Tripathi et al. (2020) demonstrated that cultures initiated at 0.5% parasitaemia peaked at ∼15% by days 4–5, followed by a crash with the emergence of early gametocytes on days 6–7. In a culture-adapted strain of *P. knowlesi* H, Armistead et al. (2018) showed that the crash method did not lead to a marked increase in gametocyte production. However, the highly synchronized *in vitro* cultures resulted in mosquito infections even when gametocyte concentrations are low or sub-microscopic.

**TABLE 1:** Studies on stress induction in gametocytogenesis.

Study	Parasite species and strain(s) used	Gametocyte induction method(s)	Gametocyte yield outcome	References
Enhanced gametocyte formation *in vitro* in reticulocyte-rich blood	*P. falciparum* HB-3 clone	50% suspension of reticulocyte-rich (immature erythrocytes) blood.	All reticulocyte-enriched blood samples yielded markedly elevated quantities of gametocytes relative to normal control blood, with gametocytes as a percentage of asexual forms being up to tenfold higher in reticulocyte-rich blood.	Trager et al., 1999
Improved synchronous production of *Plasmodium falciparum* gametocytes *in vitro*	*P. falciparum* 3D7A	Sudden increase in haematocrit on day 2, with optimal parasitaemia of 8 and 10%, combined with partially spent medium.	The protocol simplified the gametocytogenesis stimulation process, enabling synchronous gametocyte production. Although up to 5 × 10^7^ stage V gametocytes can be produced per flask, batch-to-batch irregularities remain inevitable.	Fivelman et al., 2007
A high-throughput screen targeting malaria transmission stages opens new avenues for drug development	*P. falciparum* P2G12 164/GFP	Haematocrit was reduced from 4 to 2% after 24 h by adding the culture with fresh medium.	The sexual stages (GFP-positive subpopulation of infected erythrocytes) grew from background levels of ∼ 0.005% to ∼ 0.15% at day 2, showing a 30-fold absolute increase or a 5-fold relative increase when correcting for replication rate.	Buchholz et al., 2011
Comparison and optimization of different methods for the *in vitro* production of *Plasmodium falciparum* gametocytes	*P. falciparum* 3D7, Dd2, NF54, FCR3, W2, HB3	Lysed uninfected erythrocytes (LUE) with sorbitol synchronization; 0.75% parasitaemia, 2% hematocrit, LUE (20%) was added on day 4.	NF54 produced complete stage I-V gametocytes with high reproducibility, achieving a gametocytaemia of 1.18% ± 0.07, which was significantly higher than control experiments that did not include LUE or sorbitol treatment (*p* = 0.0034).	Roncalés et al., 2012
Nowhere to hide: interrogating different metabolic parameters of *Plasmodium falciparum* gametocytes in a transmission blocking drug discovery pipeline towards malaria elimination	*P. falciparum* NF54, 3D7, FCR3, W2, HB3, and 7G8	Nutrient starvation combined with a drop in haematocrit: parasites were exposed to glucose-free medium followed by haematocrit drop from 6 to 3%.	The gametocytogenesis induction protocol was optimized, resulting in an average gametocytaemia of 5.1% on day 11 and a conversion factor of ∼33% of asexual parasites committing to gametocytogenesis. After enrichment to >95% purity, ∼677,500 ± 83,815 mature stage IV-V gametocytes were produced per mL of original culture volume.	Reader et al., 2015
Large-scale production of *Plasmodium falciparum* gametocytes for malaria drug discovery	*P. falciparum* *NF*54^*pfs*16−*LUC*−*GFP*^	Drop in haematocrit from 5 to 1.25%, combined with a sudden increase in parasitaemia to 3% trophozoites, adding 25% conditioned medium, and introducing RBC lysis products from ruptured synchronous schizonts.	Starting from an initial asexual blood-stage culture of 10 mL, the protocol yielded between 500 million and 1,000 million stage IV gametocytes within 16 days. The efficiency of induction achieved an average of 2.3 million gametocytes per mL of day 3 culture across multiple inductions performed over a 2-year period. Notably, two large inductions conducted on 27-10-2013 yielded 1,313 million and 750 million gametocytes, showing a 40% yield difference despite maintaining high induction efficiencies.	Duffy et al., 2016
Lysophosphatidylcholine regulates sexual stage differentiation in the human malaria parasite *Plasmodium falciparum*	*P. falciparum* Pf2004/164tdTom	Serum-free medium (-SerM) supplemented with 0.39% fatty acid-free bovine serum albumin together with minimal fatty acids (oleic and palmitic acid) were used. Tightly synchronized parasites (0.3–0.5% parasitaemia and 2.5% haematocrit) were incubated with the -SerM for 22 h. Media with serum (+SerM) were used post 22 h testing phase and medium was changed daily.	Sexual differentiation rates reached approximately 30% in -SerM condition compared to significantly lower rates in +SerM condition (> 2%).	Brancucci et al., 2017
The development of sexual stage malaria gametocytes in a wave bioreactor	*P. falciparum* 3D7, FCR3	Nutrient depletion: parasites with 0.8 to 1.2% parasitaemia, 6% hematocrit are cultured in a wave bioreactor™ 20/50 EHT culture system without medium change to accelerate glucose depletion.	The optimal growth conditions allowed a 1-L culture to produce up to 100 million mature stage V gametocytes in 14–16 days, making the wave bioreactor a practical and low-maintenance method to induce gametocytogenesis.	Demanga et al., 2017
Infection of mosquitoes from *in vitro* cultivated *Plasmodium knowlesi* H strain	*P. knowlesi* PkH/FZ8	Crash method and a drop in haematocrit: cultures are initiated at 0.2% haematocrit in rhesus macaque serum. Progressive haematocrit drop via daily 75% medium replacement without addition of RBCs, leading to asexual population collapse (“crash”) at 5–12% parasitaemia.	Despite the low densities, *P. knowlesi* can produce infectious gametocytes *in vitro*. In particular, *in vitro* cultures produced irregularly detected gametocytes at rates as high as 0.05%, whereas mosquito infections showed gametocyte densities ranging from 0.01 to 0.1%.	Armistead et al., 2018
Polyunsaturated fatty acids promote *Plasmodium falciparum* gametocytogenesis	*P. falciparum* NF54 and 3D7	Polyunsaturated fatty acids (PUFA) supplementation: cultures initiated at 0.1% parasitaemia and 6% haematocrit were maintained at 3% haematocrit from day 3. Small unilamellar vesicles (SUVs) containing phospholipids were added daily.	SUVs that contain equal amounts of phosphatidylcholine and phosphatidylethanolamine with 18:0–20:4 (arachidonic acid) and 18:0–22:6 (docosahexaenoic acid) raised gametocytaemia to ∼3%, comparable to 10% human serum. Meanwhile, SUVs with 16:0–18:1 (palmitoleic acid) had decreased gametocytaemia levels of 1.5 – 2.0%. The statistical analysis indicated that some PUFA-supplemented cultures had significantly higher gametocytaemia levels than those with AlbuMAX alone.	Tanaka et al., 2019
Artemisinin exposure at the ring or trophozoite stage impacts *Plasmodium falciparum* sexual conversion differently	*P. falciparum* NF54-gexp02-Tom, E5-gexp02-Tom, and NF54-10.3-Tom lines	Dihydroartemisinin (DHA) and Chloroquine (CQ): tightly synchronized cultures with 3% haematocrit and 1.5% parasitaemia were exposed to 5 nM DHA for 3 h at 22 h post-synchronization, followed by 50 mM of GlcNac and 10% human serum for 4 days; CQ with concentration range 10–160 nM was also evaluated.	Exposure of tropozoite-stage parasites to a 5 or 10 nM DHA pulse enhanced sexual conversion rates, increasing from <10% in control cultures to ∼40%. CQ is also found to enhance sexual conversion rate for about twofold (lower compared to DHA) in trophozoites. However, exposure of both drugs to the trophozoite stage did not increase sexual conversion in choline-depleted media. On the other hand, no increase was observed when ring stages were exposed to both the DHA and CQ.	Portugaliza et al., 2020
Lactic acid supplementation increases quantity and quality of gametocytes in *Plasmodium falciparum* culture	*P. falciparum* NF54	Lactic acid supplementation: the parasites were maintained in 4% haematocrit and 0.5% parasitaemia, with medium replaced every 24 h. The culture was continuously supplemented with 8.2 mM lactic acid.	When compared to control cultures that did not receive any supplementation, the quantity of gametocytes increased significantly with continuous lactic acid supplementation, achieving a 5.91-fold increase by day 5 and a 3.13-fold increase by Day 15 (*P* < 0.0001). For day 0–8 lactic acid supplementation, gametocytaemia increased by ∼4.93-fold by day 5 compared to controls. However, by day 15, this increase was reduced to only 2.3-fold (*p* = 0.0009). In contrast, lactic acid supplementation on days 9–16 and days 8–9 did not result in a statistically significant increase in gametocyte quantity relative to controls (*P* < 0.05).	West and Sullivan, 2020
Investigation of factors affecting the production of *P. falciparum* gametocytes in an Indian isolate	*P. falciparum* RKL-9 Indian isolate	Young erythrocytes (0–7 days post-collection) and hypoxanthine supplementation (50–100 mg/L): cultures initiated at 0.5% parasitaemia and 10% haematocrit. The haematocrit was reduced by half on day 8 with fresh medium added and without adding more erythrocytes.	Adding hypoxanthine significantly increased the production of gametocytes, particularly at a concentration of 50 mg/L, which led to a mean gametocytaemia of 2.14% ± 0.14 as opposed to 0.81% ± 0.05 when hypoxanthine was not present (*p* < 0.05). Furthermore, gametocytaemia increased significantly (2.24% ± 0.15) in the 100 mg/L hypoxanthine group. The study showed that fresh erythrocytes (0–7 days post-collection) had a significantly greater gametocytaemia of 2.14% ± 0.14 than older erythrocytes (15–20 days post-collection) with a gametocytaemia of 0.79% ± 0.25, at *p* < 0.05.	Wadi et al., 2021
Simple supplementation of serum-free medium produces gametocytes of *Plasmodium falciparum* that transmit to mosquitoes	*P. falciparum* 3D7	Serum-free medium (-SerM) supplemented with 0.5% AlbuMAX II only (CM-A) or 0.5% AlbuMAX II together with insulin, transferrin, sodium selenite, and ethanolamine (CM-A + ITS-X) were used. Parasites were maintained at 0.2% parasitaemia and 5% haematocrit using the respective media, and fresh media were replaced daily up to day 6. Then, 15-17 day old gametocytes were fed to *An. gambiae* via membrane feeding.	Both CM-A and CM-A + ITS-X produced significantly higher gametocyte numbers than medium supplemented with 10% serum (*P* = 5.5 × 10^–6^ and *P* = 0.00039, respectively). Besides, CM-A + ITS-X improved the mosquito infectivity compared to CM-A with higher infection prevalence and oocyst number (model predictions).	Pradhan et al., 2024

Over the years, many researchers used a parasite-conditioned medium (CM) as the technique for triggering gametocytogenesis. CM is derived from a high-parasitaemia culture (Brancucci et al., 2015), which was hypothesized to be either deprived of particular host components or enriched in particular secreted parasite factors (Brancucci et al., 2017). According to a study led by Brancucci et al. (2015), the addition of CM dramatically boosts the rate of gametocyte yield, with expected conversion rates increasing from ∼ 0.3% to 14%–24%. Based on their protocol, CM collected from cultures achieving parasitaemia levels between 5.5 and 6.5% was most effective for gametocytogenesis when used at a 90% (v/v) working concentration. Meanwhile, the large-scale gametocyte production in *P. falciparum* has been shown by Duffy et al. (2016) utilizing a combination of fresh media and spent media from the parasite culture. When ring-stage parasitaemia reached 10%–12% on day 2 of gametocyte induction, their procedure recommended resuspending the culture in a medium consisting of 75% fresh asexual culture medium and 25% conditioned (spent) material. In contrast to Brancucci et al. (2015), who primarily relied on CM to obtain high conversion rates of 14%–24%, Duffy et al. (2016) used CM in conjunction with other stressors, including decreased haematocrit levels, elevated parasitaemia, and RBC lysis products, to produce significantly higher absolute gametocyte yields, in the hundreds of millions starting from a 10 mL culture. Whereas Brancucci’s work underscored the efficiency of CM in improving sexual conversion, Duffy’s work showcased its scalability for mass gametocyte production. Human serum has long been used in culture systems, although the use is constrained by its high cost, variability, and ABO compatibility problems (Pradhan et al., 2024). The latest research showed that AlbuMAX™ II (a lipid-rich bovine serum albumin) supplemented with ITS-X (insulin, transferrin, sodium selenite, and ethanolamine) is able to produce *P. falciparum* 3D7 gametocytes in a serum-free medium (-SerM). This combination not only improved mosquito infectivity in *Anopheles gambiae* but also increased gametocyte yield, as media containing AlbuMAX™ II as well as AlbuMAX™ II + ITS-X produced significantly higher gametocyte numbers than 10% serum cultures. A number of factors, such as insulin, transferrin, and selenium, are likely responsible for the effect, as they contribute to the growth of asexual parasites (Pollack and Fleming, 1984; Gamain et al., 1996; Balaji et al., 2020; Pradhan et al., 2024). Additionally, ethanolamine, a component of membrane phospholipids, is likely to be essential for the production of transmission-competent gametocytes, which exhibit higher lipid requirements compared to asexual stages (Gulati et al., 2015; Tran et al., 2016; Brancucci et al., 2017; Pradhan et al., 2024). The findings reported by Pradhan et al. (2024) were supported by a similar study by Brancucci et al. (2017), as they unveiled that substituting human serum with fatty acid-free bovine serum albumin together with minimal fatty acids (oleic and palmitic acid) in a serum-free medium led to an increase in gametocyte production across multiple *P. falciparum* strains, as sexual differentiation rates reached approximately 20%–30% in -SerM conditions, compared to significantly lower rates in +SerM conditions. Therefore, the serum-free approach provides a more affordable and dependable alternative for parasite transmission studies (Pradhan et al., 2024).

Lactic acid supplementation was used in a study by West and Sullivan (2020) to promote gametocytogenesis in low-passage-number NF54. Erythrocytes are able to produce lactic acid independently, and high levels of lactic acid have been detected in the bone marrow of malaria patients (Seheult et al., 2017; Possemiers et al., 2021). Notably, parasites alone can produce millimolar quantities of lactic acid within 24 h *in vitro*, with the production peaks during asexual phases and decreases as gametocytes mature (Lamour et al., 2014). Lactic acid concentrations above 16 mM may act as a stress signal that enables parasites to detect changes in their environment, preventing asexual parasites from replicating (Zolg et al., 1984; Hikosaka et al., 2015). By adding a final concentration of 8.2 mM D, L-lactic acid to the culture media at various time points and duration, West and Sullivan (2020) discovered that daily culture medium exchange with continuous 8.2 mM lactic acid supplementations (day 0–16) significantly increased gametocytes density after 5 days in comparison to supplementations given during the first half of culturing (days 0–8), the second half (days 9–16), and the short pulse (days 8–9).

LUE suspension mimics the accumulation of erythrocyte debris and hemoglobin-depleted erythrocytes seen in patients after high asexual parasite loads, with free hemoglobin potentially serves as one of the unknown factors triggering sexual differentiation *in vitro* (Schneweis et al., 1991; Bennett et al., 2005; Roncalés et al., 2012). Building on this, Roncalés et al. (2012) combined both crucial stressors, with 20% (v/v) LUE supplementation and complete elimination of asexual stages via sorbitol treatment to create an optimal protocol using NF54 parasites. This method resulted in the highest, fully staged (I-V) gametocytaemia with remarkable reproducibility. Similarly, Wadi et al. (2021) prepared LUE suspension using a cold-heat shock method and introduced it to RKL-9 *P. falciparum* culture 48 h post-culture initiation. No significant difference was observed in the final gametocytaemia between the LUE-treated culture compared to the control. This result is contradicted with the previous finding by Schneweis et al. (1991), in which production of gametocytes is enhanced by LUE. Thus, Wadi et al. (2021) proposed that parasites’ ability to produce and develop gametocytes may differ in strains despite the same induction approach being used. They further showed that supplementing RKL-9 gametocyte cultures with hypoxanthine significantly increased gametocyte yield and supported healthy maturation through stage V.

Several studies indicated that exposure to antimalarial drugs may increase the parasite’s commitment to gametocytogenesis, despite the lack of conclusive evidence (Price et al., 1996; Barnes et al., 2008). It has been proposed that stress induced by drugs may cause a “terminal investment” response, in which *Plasmodium* shifts to gametocyte production when their survival is threatened. This idea is further supported by Portugaliza et al. (2020), who showcased that exposure to dihydroartemisinin (DHA), an important artemisinin derivative, impacts sexual conversion differently depending on the parasite stage. In particular, DHA treatment at 5 nM and 10 nM during trophozoite-stage markedly increased sexual commitment, rising from less than 10% to ∼40% in cultures supplemented with choline. In contrast, DHA treatment applied during the ring stage decreased both gametocytaemia and sexual conversion. Studies on stress induction in gametocytogenesis are summarized in Table 1.

## 4 Genetic manipulation approaches in understanding gametocytogenesis

While environmental stressors or culture adaptation provides observational understandings on gametocytogenesis, molecular analysis offers critical insights into its fundamental regulation. Gene editing especially, enables precise functional dissection of regulatory genes, thus useful in understanding malaria transmission and targeting important genes. One of the earliest methods applied was double homologous recombination, where plasmid constructs with long homology arms flanking a selectable marker are introduced by electroporation (Janse et al., 2006). This method was key for targeted gene disruption, as demonstrated by Kafsack et al. (2014), who generated *ap2-g* knockout *P. falciparum* parasites. The mutants were unable to produce gametocytes, identifying PfAP2-G as a central regulator of sexual commitment. In the FKBP-destabilization domain system, PfAP2-G stability was made dependent on the ligand Shield-1, and its graded depletion led to proportional decrease in gametocyte formation. Despite its precision, this approach has technical limitations, including low transfection efficiency in *P. falciparum*, the need for laborious cloning and selection, and the inability to scale easily for genome-wide studies.

PiggyBac transposon-mediated insertional mutagenesis has also been employed in the past (Ikadai et al., 2013). By co-transfecting parasites with a transposon and a transposase-encoding plasmid, they generated random insertional mutants across the genome. Screening 189 drug-resistant clones, they identified 29 mutants that failed to form mature gametocytes. Mapping these insertions revealed disruptions in 16 genes, including an AP2 transcription factor, ribosomal proteins, RNA processing factors, and several genes of unknown function. Complementation of five piggyBac insertional mutants (2A2, 2A11, 2G2, 2G11, and 2F4) restored gametocyte development, confirming causality. Nonetheless, the piggyBac approach has notable drawbacks, which are random insertion making gene targeting unpredictable, essential genes in asexual stages generally absent due to lethality, and multiple insertions in single clones complicating phenotype interpretation.

On the other hand, drug selection has been used to isolate spontaneous resistant mutants (Goodman et al., 2016). By culturing parasites in the presence of atovaquone, which targets mitochondrial cytochrome b (cytB), they obtained parasites with point mutations in cytB. While these mutants grew normally in asexual blood stages and did produce gametocytes, they failed to complete development in mosquitoes, thus blocking transmission. Genetic crossing experiments confirmed that the defect was maternally inherited via the female gamete. This strategy effectively linked mitochondrial function to gametocyte transmission competence. However, selection-based methods are inherently restricted to phenotypes under drug pressure and carry the risk of selecting secondary mutations unrelated to the targeted pathway.

The aforementioned methods were eventually surpassed by CRISPR/Cas9 technology, which enables targeted double-strand breaks, greatly enhances editing efficiency, and allows for more precise and versatile genetic modifications. Most of the works focus on GDV1 as it regulates HP1 and *ap2-g* expressions. Filarsky et al. (2018) applied CRISPR/Cas9 to tag the endogenous *gdv1* gene with a 3 × HA epitope, allowing precise visualization of GDV1 protein dynamics under environmental cues. Their work clearly demonstrated that GDV1 disrupts HP1-mediated silencing at the *ap2-g* locus, lifting the repression of this master regulator and triggering sexual commitment. Importantly, they linked metabolic triggers like choline depletion to GDV1 upregulation, effectively connecting environmental sensing to genetic control. Next, Tibúrcio et al. (2021) generated a C-terminal truncation mutant, GDV1Δ39 via conditional knockout system. Although GDV1Δ39 retained nuclear localization and could still interact with HP1, it was unable to effectively induce *ap2-g* expression or drive sexual commitment. The critical reason was that the truncated GDV1 protein was present at insufficient levels to reach the induction threshold required for sexual commitment, despite its preserved targeting functions. Boltryk et al. (2021) engineered inducible gametocyte producer (iGP) lines by inserting a conditional GDV1 overexpression cassette into the *cg6* locus. This system, controlled by Shield-1, consistently induced ∼75% sexual conversion and synchronous gametocyte production, generating robust, transmission-competent parasites. The iGP lines provided a powerful platform to study late-stage gametocyte biology and evaluate transmission-blocking interventions.

Bui Hai et al. (2021) shifted focus to HP1, employing CRISPR/Cas9 with DiCre/loxP-mediated conditional deletions to dissect domain-specific functions. Their results showed that loss of any HP1 domain; chromodomain, hinge, or chromoshadow led to collapse of heterochromatin and massive derepression of *ap2-g*, causing near-total conversion to gametocytogenesis. This firmly positioned HP1 as an indispensable epigenetic gatekeeper in the parasite’s developmental fate. On the other hand, Liang et al. (2022) expanded the toolkit by introducing an inducible CRISPR interference/activation (CRISPRi/a) system, using dCas9 fused to GCN5 or Sir2a to epigenetically modulate gene expression without introducing permanent mutations. Notably, they achieved >10-fold induction of *ap2-g* and a fourfold increase in gametocyte formation, all under tight rapamycin-inducible control via DiCre/loxP. This system allowed reversible, stage-specific gene regulation, even in gametocytes, providing a highly versatile and fine-tuned approach for dissecting gene function, especially in essential or developmentally regulated loci.

Looking into other target genes, Tay et al. (2016) approached the problem from the angle of post-translational regulation, disrupting the palmitoyl transferase P fDHHC9 via gene knockout. While asexual growth remained unaffected, gametocyte formation was markedly impaired, implicating palmitoylation as a novel regulatory layer in sexual differentiation and opening avenues for exploring lipid modification pathways as potential transmission-blocking targets. Wezena et al. (2017) used CRISPR/Cas9 to disrupt the cytosolic glyoxalase genes *PfGlo1* and *PfcGlo2* . While knockout of either gene did not impair the asexual growth, loss of *PfcGlo2* significantly increased gametocyte commitment. This unexpected finding suggests that the accumulation of S-D-lactoylglutathione or related metabolic intermediates may act as stress signals or internal cues tipping the balance toward sexual differentiation, revealing a previously understudied metabolic link in the regulation of commitment.

## 5 Conclusion

The diverse strategies employed to induce gametocytogenesis highlight the complexity of this process and the need for further mechanistic understanding. Although stress-based methods remain essential, they do not fully replicate the physiological triggers that drive transmission in natural infections, limiting their predictive value for transmission-blocking studies. Molecular approaches, on the other hand, have advanced our understanding of regulatory networks governing sexual commitment, yet this knowledge has yet to yield practical tools for large-scale gametocyte production across *Plasmodium* species. To achieve large-scale gametocytogenesis, it is imperative to bridge the gap between mechanical and molecular methods, leveraging insights from one domain to refine the other. Such advances have direct implications for global malaria control. Reliable large-scale gametocyte production is not only critical for accelerating the discovery of transmission-blocking drugs, but also indispensable for vaccine development strategies that rely on mosquito infection and sporozoite harvesting. Furthermore, genome editing platforms that fine-tune gametocyte induction provide powerful systems to test candidate interventions under physiologically relevant conditions. Ultimately, translating our understanding of gametocytogenesis into practical laboratory platforms will unlock a pipeline of therapeutic and vaccine innovations, bringing the goal of malaria elimination closer to reality.
